# Life-Course Programming of Kidney Disease: Roles of Gut Microbiota Dysbiosis and Oxidative Stress

**DOI:** 10.3390/antiox15060707

**Published:** 2026-06-03

**Authors:** Chien-Ning Hsu, You-Lin Tain

**Affiliations:** 1 Department of Pharmacy, Kaohsiung Municipal Ta-Tung Hospital, Kaohsiung 801, Taiwan; cnhsu@cgmh.org.tw; 2 School of Pharmacy, Kaohsiung Medical University, Kaohsiung 807, Taiwan; 3 Department of Pharmacy, Kaohsiung Chang Gung Memorial Hospital, Kaohsiung 833, Taiwan; 4 Department of Pediatrics, Kaohsiung Chang Gung Memorial Hospital, Kaohsiung 833, Taiwan; 5 College of Medicine, Chang Gung University, Taoyuan 333, Taiwan; 6 Doctoral Program of Clinical and Experimental Medicine, National Sun Yat-Sen University, Kaohsiung 804, Taiwan

**Keywords:** gut microbiota, oxidative stress, antioxidants, chronic kidney disease, hypertension, developmental origins of health and disease (DOHaD), probiotics, prebiotics, life-course approach

## Abstract

Chronic kidney disease (CKD) affects millions globally and represents a major health burden. This narrative review adopts a life-course perspective to synthesize current evidence on CKD as a consequence of adverse early-life exposures that disrupt nephrogenesis, leading to kidney programming and reduced nephron endowment. The objective of this review is to integrate emerging mechanistic and translational evidence linking developmental programming, gut microbiota, and redox biology within a unified gut–redox axis framework, and to identify potential targets for early-life prevention of CKD. Central to this process is the gut–redox axis, a bidirectional network linking gut microbiota with host redox homeostasis. A balanced axis preserves epithelial integrity, metabolic stability, and immune regulation, whereas dysbiosis and oxidative stress form a self-perpetuating cycle that promotes CKD and related comorbidities. Maternal oxidative stress and impaired microbial transmission exacerbate early-life dysbiosis, persistent epigenetic alterations, and nephron deficits. In adulthood, protein-bound uremic toxins amplify oxidative injury and inflammation, further perturbing microbial composition. Experimental and clinical studies show that early-life interventions—including probiotics, prebiotics, postbiotics, antioxidants, and toxin-lowering strategies—can restore gut–redox balance and improve renal outcomes. These insights highlight opportunities for precision prevention and mechanism-based therapies targeting CKD across the life course.

## 1. Introduction

Globally, chronic kidney disease (CKD) affects over 700 million individuals—approximately 10% of the adult population—and ranks among the leading causes of death and disability, representing a significant and escalating public health burden [1]. While traditional nephrology has historically focused on the cumulative impact of sequential renal injuries in adulthood, emerging evidence necessitates a paradigm shift toward a life-course approach grounded in the Developmental Origins of Health and Disease (DOHaD) context [2]. This perspective recognizes CKD as a multifactorial disorder rooted in developmental programming, whereby adverse exposures during critical windows of developmental plasticity—particularly the first 1000 days from conception to the child’s second birthday—shape long-term renal structure, function, and disease susceptibility [3]. Adverse maternal conditions—including nutritional imbalance, metabolic disturbances such as gestational diabetes mellitus (GDM), and exposure to environmental toxicants (e.g., heavy metals and phthalates)—can disrupt fetal nephrogenesis, leading to persistent structural and functional alterations via a process referred to as “kidney programming” [4]. A congenitally reduced nephron endowment constitutes the definitive “first hit,” which lowers the threshold for kidney failure when the host is later subjected to environmental or metabolic stressors [5,6].

At the center of this lifelong disease trajectory is the gut–redox axis, a complex, bidirectional regulatory network linking the intestinal microbial ecology with host redox homeostasis [7,8,9]. Redox status—the equilibrium between pro-oxidants, primarily reactive oxygen and nitrogen species (ROS/RNS), and the host’s antioxidant defense systems—is a fundamental modulator of life and kidney health [10,11]. A balanced axis maintains intestinal epithelial barrier integrity, promotes systemic metabolic stability, and regulates immune signaling pathways [12]. In a state of eubiosis, diverse microbial communities produce beneficial metabolites, most notably short-chain fatty acids (SCFAs) like butyrate, which strengthen tight junction proteins (e.g., ZO-1, occludin) and activate the nuclear factor erythroid 2-related factor 2 (Nrf2)/Keap1 pathway, the primary cellular defense against oxidative damage [13]. Conversely, perturbations in this axis trigger a self-perpetuating cycle of gut dysbiosis and oxidative stress [14].

Maternal oxidative stress and dysbiotic microbiota contribute to offspring kidney disease [15,16]. Accumulating human and experimental evidence indicates that the timing of gut–redox disruption critically shapes renal outcomes across the life course. Maternal redox status governs intergenerational microbial transmission. Oxidative stress induced by maternal insults disrupts placental integrity and triggers maternal immune activation, impairing vertical transfer of pioneer microbes [17,18]. Because these early colonizers help establish neonatal redox tone and immune priming, their disruption has been proposed to contribute to early-life dysbiosis and may influence epigenetic regulation and kidney developmental programming, potentially increasing susceptibility to CKD and hypertension later in life [19].

Across the life course, the gut–redox axis evolves from a developmental modulator to a central integrator of aging-related vulnerability. In adulthood—and particularly with advancing age—it functions as both mediator and amplifier of systemic stress signals. In CKD, the accumulation of protein-bound uremic toxins—such as p-cresyl sulfate (pCS), indoxyl sulfate (IS), and trimethylamine N-oxide (TMAO)—has been associated with oxidative injury and may contribute to redox imbalance and disease progression, although direct causal evidence from human interventional studies remains limited [20,21]. These toxins have been shown to be associated with increased oxidative stress through activation of inflammatory signaling pathways, including NF-κB, in a context- and cell-type–dependent manner, and may promote ROS generation via multiple sources such as mitochondrial dysfunction and NADPH oxidase (NOX) activation, thereby contributing to systemic inflammation and renal fibrotic processes. This creates a bidirectional feed-forward loop: progressive kidney impairment reshapes gut microbial ecology toward a more pathogenic profile, which generates further uremic toxins and reactive species, accelerating functional decline [22].

**Figure 1:**
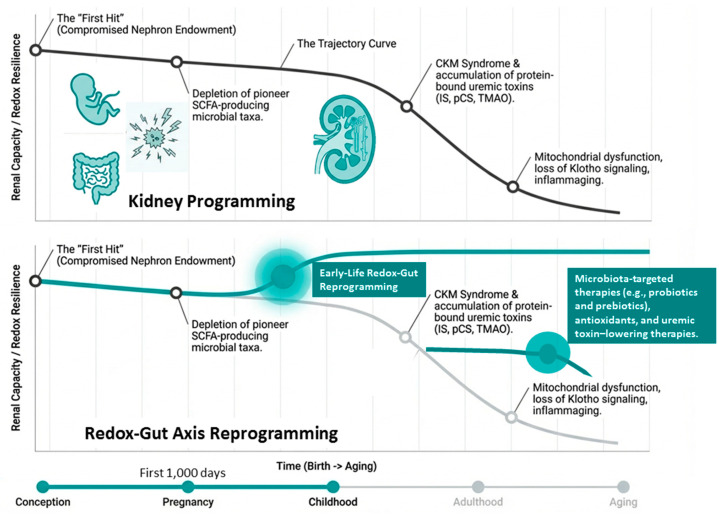
Kidney Programming Trajectory and the Effect of Early-Life Redox–Gut Reprogramming. Upper panel shows the pathological trajectory: renal capacity declines from conception after a prenatal “first hit” reduces nephron endowment. Early loss of SCFA-producing microbiota accelerates deterioration, followed by adult accumulation of uremic toxins (IS, pCS, TMAO) leading to cardiovascular-kidney-metabolic (CKM) syndrome, and later mitochondrial dysfunction, Klotho deficiency, and chronic inflammation. Lower panel shows the therapeutic trajectory: early-life Redox–Gut reprogramming within the first 1000 days shifts the curve toward preserved renal resilience. Microbiota-targeted therapy, antioxidants, and toxin-lowering strategies restore redox balance, maintain kidney function, and delay age-related decline.

Clinical and preclinical evidence indicates that this axis is modifiable. Microbiota-targeted interventions (e.g., probiotics and prebiotics), redox-modulating antioxidants, and uremic toxin–lowering therapies have demonstrated the potential to attenuate oxidative stress, restore microbial homeostasis, and improve renal outcomes [23,24,25], particularly when implemented early in life through reprogramming strategy [4,19]. By integrating findings from human and experimental research, this narrative review defines the mechanistic framework of gut–redox interactions across the life course and highlights emerging opportunities for precision prevention and mechanism-based interventions. The overall concept is summarized in Figure 1.

## 2. Materials and Methods

Given the conceptual breadth of the life course framework and the heterogeneity of experimental and clinical methodologies in this field, this review was conducted using a narrative synthesis approach designed to facilitate theory-driven integration rather than quantitative evidence grading. This strategy enabled the consolidation of evolving mechanistic insights across developmental biology, nephrology, microbiome science, redox biology, and aging research within a unified gut–redox–kidney axis framework.

A structured literature search was conducted to identify peer-reviewed articles published in English between January 2000 and December 2025. Major biomedical databases, including MEDLINE, Embase, and the Cochrane Library, were systematically queried. Study selection followed a two-stage screening process: (i) title and abstract screening for relevance, and (ii) full-text assessment for eligibility based on predefined inclusion criteria.

The search strategy was organized around four conceptual modules aligned with the life course model: (1) developmental programming and DOHaD-related kidney vulnerability (e.g., maternal exposures, placental dysfunction, nephrogenesis, early-life insults, epigenetic regulation); (2) gut microbiota–redox signaling interactions (e.g., ROS/RNS pathways, Nrf2/Keap1 signaling, NF-κB activation, gut microbiota composition, microbial metabolites, immune modulation); (3) uremic toxins, inflammaging, and CKD progression (e.g., indoxyl sulfate, TMAO, fibrosis, cellular senescence); and (4) reprogramming and therapeutic modulation strategies targeting microbial ecology or redox balance.

Eligibility criteria included: (i) original experimental or clinical studies, (ii) studies addressing at least one component of the gut–redox–kidney axis, and (iii) articles providing mechanistic, translational, or clinical insight relevant to kidney development, CKD progression, or intervention. Exclusion criteria were: (i) non-peer-reviewed literature, (ii) non-English publications, (iii) studies not directly related to renal outcomes or gut–redox mechanisms, and (iv) purely descriptive reports without mechanistic or clinical relevance.

Both human studies and experimental animal models were included to enable cross-species translational interpretation, with greater interpretative weight given to human data where available, while animal studies were used primarily to support mechanistic insight rather than direct clinical extrapolation, particularly given known interspecies differences in gut microbiota composition and metabolic function. Inclusion priority was given to high-quality mechanistic studies, clinically relevant translational research, and landmark publications that significantly contributed to the DOHaD or gut–kidney axis framework. Reference lists of selected articles were manually screened to ensure comprehensive coverage of seminal and emerging studies. This concept-oriented methodology allowed for a cohesive evaluation of mechanistic continuity from early-life programming to aging-associated renal decline, highlighting modifiable nodes within the gut–redox axis relevant to precision prevention and intervention. During manuscript preparation, the authors used ChatGPT (version GPT-5.4 mini) to improve language clarity and readability and used NotebookLM (Google) and Gemini 3.0 to assist in figure organization. The authors carefully examined and revised all generated content and take full responsibility for the correctness and integrity of the final version.

## 3. Bidirectional Interactions Between Gut Microbiota and Redox Signaling

**Figure 2:**
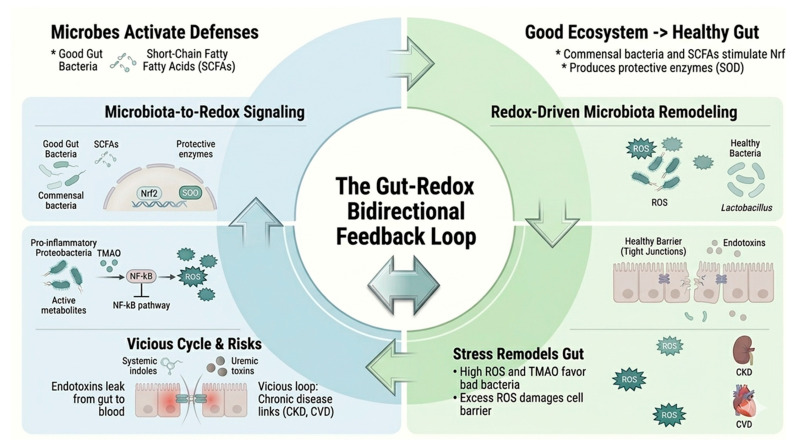
Bidirectional Gut–Redox Feedback Loop in Health and Disease. This figure illustrates the dynamic reciprocal interaction between gut microbiota and host redox homeostasis, showing a virtuous cycle in health (top/right) and a vicious cycle in disease (bottom/left).

Redox status refers to the dynamic balance between the generation of ROS/RNS and the host’s antioxidant defense systems that maintain cellular homeostasis [10]. Within the gastrointestinal ecosystem, this balance is powerfully influenced by the intestinal microbiota, giving rise to the concept of the gut–redox axis—a bidirectional network linking microbial ecology, host oxidative signaling, and immune regulation [14,26]. Through this axis, microbial composition and metabolic activity shape the oxidative environment of the intestine and influence systemic physiological processes (Figure 2).

### 3.1. Microbiota-to-Redox Signaling

The gut microbiota regulates host redox homeostasis through two principal signaling pathways. Dysbiotic microbial communities are often associated with shifts in community composition, which may include expansion of certain Gram-negative taxa such as members of the *Proteobacteria* phylum in specific contexts, potentially increasing luminal lipopolysaccharide (LPS) burden. LPS activates the Toll-like receptor 4 (TLR4)/NF-κB signaling cascade, promoting pro-inflammatory cytokine production and NADPH oxidase-mediated ROS generation, thereby amplifying oxidative stress and inflammation [27,28]. In contrast, commensal microbes including *Lactobacillus* and *Bifidobacterium* stimulate the Nrf2/Keap1 antioxidant pathway, inducing transcription of protective enzymes such as superoxide dismutase (SOD), catalase (CAT), and glutathione peroxidase (GPx) that restore redox equilibrium [29].

Microbial metabolites are key regulators of the gut–redox network. SCFAs (acetate, propionate, butyrate) inhibit histone deacetylases and activate GPCR signaling, thereby upregulating Nrf2 and enhancing antioxidant enzymes (e.g., SOD, CAT), which reduces oxidative stress [30,31]. In contrast, dysbiosis promotes accumulation of deleterious metabolites such as TMAO and IS, which exacerbate mitochondrial dysfunction, oxidative stress, and tissue injury. TMAO, derived from dietary choline and carnitine, impairs mitochondrial function and activates the NLRP3 inflammasome, promoting renal fibrosis and further perturbing redox homeostasis. These processes collectively accelerate endothelial senescence and vascular aging [32]. IS and pCS, which are protein-bound and poorly cleared by dialysis, enter renal tubular cells via organic anion transporters (OATs) and induce oxidative stress through multiple complementary pathways, including NF-κB activation, AhR signaling, and promotion of tubular cellular senescence, leading to increased ROS production and renal injury [33]. Additionally, tryptophan-derived indole metabolites support epithelial barrier integrity via AhR signaling [34]. Notably, AhR acts as a ligand-dependent redox regulator, whereby uremic toxins promote oxidative stress whereas beneficial microbial metabolites enhance antioxidant defenses, linking gut microbial metabolism to host redox homeostasis [35].

### 3.2. Redox-Driven Remodeling of the Gut Microbiota

Oxidative stress is a key ecological force shaping the gut microbiota [26]. ROS from mitochondrial leakage, immune activation, or environmental exposures alter the intestinal redox environment and damage microbial components, favoring expansion of facultative anaerobes and pro-inflammatory taxa such as *Proteobacteria* (e.g., *Escherichia coli*) [36], while suppressing beneficial anaerobes (*Bacteroidetes*, *Lactobacillus*, *Bifidobacterium*). This shift reduces SCFA production and further disrupts intestinal homeostasis.

Redox signaling pathways mediate these microbiota shifts. ROS activates the NF-κB pathway, promoting pro-inflammatory cytokine release that disrupts microbial balance, whereas impairment of the Nrf2 antioxidant pathway weakens mucosal defenses and allows oxidative conditions that favor pathogenic bacteria [37]. Dysbiosis, in turn, increases LPS-producing Gram-negative bacteria, which stimulate TLR4/MyD88 signaling, further amplifying inflammation and ROS generation [28]. Together, these processes constitute a bidirectional feedback loop rather than a unidirectional cascade. Excess ROS contributes to disruption of epithelial tight junction integrity, including ZO-1, occludin, and claudins, both through direct oxidative modification and indirectly via activation of kinase-dependent pathways such as MLCK-mediated phosphorylation, thereby increasing intestinal permeability and facilitating endotoxin translocation. Together, these processes form a self-reinforcing cycle in which oxidative stress reshapes the microbiota, and dysbiosis amplifies oxidative and inflammatory signaling, linking the gut–redox axis to CKD and related cardiometabolic diseases.

## 4. Gut–Redox Axis in Pregnancy and Fetal Development

Recent research increasingly highlights the gut–redox axis as an important pathway influencing maternal biological regulation and the programming of fetal development [38,39]. During pregnancy, the maternal gut microbiota undergoes dynamic remodeling to accommodate the metabolic demands of gestation and fetal growth. These microbial shifts interact with hormonal regulation, nutrient metabolism, and immune signaling, collectively shaping the maternal redox environment that influences fetal development and long-term disease susceptibility [40,41].

### 4.1. Redox Status and Gut Remodeling in Normal Pregnancy

Pregnancy triggers coordinated hormonal, metabolic, and immunological adaptations to support fetal growth and maternal energy balance. Maternal gut microbiota undergoes substantial restructuring [42], and this period is characterized by localized, tightly regulated ROS signaling [43,44], which supports fetal nourishment and energy storage for lactation. Nitric oxide (NO) plays a dual role in redox biology: under physiological conditions, it maintains vascular homeostasis and antioxidant balance, whereas under oxidative stress it reacts with superoxide to form peroxynitrite, a highly reactive species that promotes nitrosative damage to lipids, proteins, and DNA while reducing NO bioavailability [45]. NO also regulates key reproductive processes, including implantation, placental angiogenesis, uterine relaxation, and fetal development [46]. Low levels of asymmetric dimethylarginine (ADMA, an inhibitor of NOS) and enhanced NO bioavailability support early gestational hemodynamic adaptation, whereas rising ADMA levels in late pregnancy modulate uterine relaxation and may contribute to the initiation of labor [47].

Longitudinal studies have demonstrated a progressive reduction in microbial richness (α-diversity) and increased interindividual variability (β-diversity) as pregnancy advances [42]. During the first trimester, the microbial composition resembles that of non-pregnant women and is typically dominated by members of the *Firmicutes* phylum. By the third trimester, however, there is a notable expansion of *Proteobacteria* and *Actinobacteria*, accompanied by a decline in butyrate-producing taxa.

These compositional shifts are partly driven by rising concentrations of pregnancy-associated hormones, particularly progesterone and estrogen. Progesterone has been shown to promote the proliferation of *Bifidobacterium* species [48]. Although the third-trimester microbiota shares certain features with dysbiotic profiles observed in metabolic syndrome, these changes are generally considered adaptive in healthy pregnancies, facilitating increased energy harvest and maternal fat storage required for lactation [42]. Nevertheless, these physiological adaptations are associated with a mild pro-inflammatory state and increased oxidative stress [49].

### 4.2. Dysregulated Gut–Redox Axis in Compromised Pregnancy

Emerging evidence identifies the gut–redox axis—the bidirectional interplay between intestinal microbial ecology and host redox signaling—as a central determinant of pregnancy outcomes and offspring programming. When this finely tuned redox balance is disrupted by maternal conditions such as obesity [50], GDM [51], preeclampsia [52], preterm birth [53], preterm premature rupture of membranes [54], or intrauterine growth restriction (IUGR) [55], pregnancy shifts from physiological stress to pathological oxidative damage.

Overweight mothers exhibit pronounced microbial dysbiosis, with decreased *Bifidobacterium* and *Bacteroides* and overgrowth of pro-inflammatory taxa such as *Staphylococcus* and *Escherichia coli* [56]. This imbalance increases LPS production and reduces beneficial metabolites, notably SCFAs like butyrate; loss of SCFA-producing bacteria such as *Faecalibacterium prausnitzii* exacerbates insulin resistance and chronic low-grade inflammation. In preeclampsia, gut microbial diversity drops further, with expansion of pathogenic taxa including *Clostridium difficile* and *Fusobacterium* [57], correlating with elevated blood pressure (BP) and renal dysfunction. Reduced SCFA production at the uteroplacental interface diminishes propionate and butyrate levels, promoting placental hypoxia and increased ROS generation.

A hallmark of compromised pregnancy is “leaky gut” [58], where pathological oxidative stress damages the intestinal barrier, allowing translocation of endotoxins and uremic precursors into the maternal circulation. Excess maternal oxidative stress may influence the fetal environment through selective placental transfer of certain redox-related molecules (e.g., H_2_O_2_) and indirect placental signaling, including inflammatory and endocrine mediators. Although not all ROS or cytokines cross the placenta freely, these pathways may contribute to fetal epigenetic programming and potentially prime offspring for long-term disease susceptibility. Understanding the shift from adaptive remodeling to a dysregulated gut–redox axis provides a mechanistic foundation for precision interventions to protect maternal and fetal health.

### 4.3. Transplacental Microbial Metabolite Signaling and Fetal Programming

The long-standing paradigm of a sterile fetal environment has been reconsidered with the recognition of a gut–placenta–fetus signaling axis, through which maternal microbial metabolites act as transplacental biochemical messengers. Although the translocation of live bacteria remains controversial [59], accumulating evidence demonstrates that numerous microbiota-derived metabolites enter the maternal circulation and cross the placenta, thereby shaping fetal development before birth [39].

Among these metabolites, SCFAs are the most extensively characterized. Produced by microbial fermentation of dietary fiber in the maternal colon, SCFAs readily reach the fetal compartment. In the fetus, they function as signaling molecules through SCFA receptors GPR41 and GPR43, promoting thymic development, regulatory T-cell differentiation, and intestinal barrier maturation [60].

Other metabolites also contribute to fetal programming. TMAO and related betaines, generated from microbial metabolism of dietary choline and carnitine, can cross the placenta and participate in fetal development. Although elevated TMAO levels are associated with cardiovascular risk in adults [61], physiological levels of maternal-derived TMAO appear to support normal fetal neurodevelopment, including processes such as thalamocortical axonogenesis [62]. In parallel, tryptophan-derived indole metabolites, including indole-3-propionate and indole-3-aldehyde, function as ligands for the AhR. These metabolites support the maturation of fetal innate immune cells and lymphoid structures while also exerting antioxidant and neuroprotective effects [63].

Importantly, the spectrum of metabolites reaching the fetus is highly sensitive to the maternal microbial state. Adverse intrauterine conditions, such as maternal high-fat diets, can induce gut dysbiosis and alter the metabolite profile available for placental transfer, often reducing beneficial SCFAs while increasing potentially harmful metabolites such as TMAO [64]. Conversely, dietary or microbiota-targeted interventions during pregnancy may enhance the production of protective microbial metabolites within the fetoplacental environment, thereby influencing fetal programming and long-term health outcomes [39,65].

## 5. CKD: A Life Course Perspective

Currently, CKD cannot be fully prevented by adult-focused strategies alone [1,2]. Although KDIGO-recommended interventions such as BP and glycemic control can reduce risk and slow disease progression, they have limited impact on the overall global burden, as they primarily target downstream manifestations rather than upstream developmental and early-life determinants of disease [25]. Nephron endowment is largely fixed before birth, and adverse intrauterine exposures can represent a “first hit” that reduces renal reserve [66]. Early-life risk factors may induce structural and functional alterations in the kidney that embed lifelong vulnerability to renal injury [67]. Therefore, integrating early-life programming into preventive strategies is essential to shift CKD management from reactive treatment toward true risk reduction.

### 5.1. DOHaD Framework

The DOHaD framework highlights how early-life exposures shape long-term kidney health [68]. Within this paradigm, kidney programming refers to impaired nephrogenesis resulting from adverse intrauterine conditions such as maternal malnutrition [69], metabolic disorders [70,71], environmental toxins [72], or medication exposure [73]. Evidence from epidemiologic studies—including famine cohorts [74]—and experimental models [75] demonstrates that insults during critical developmental windows disrupt kidney formation, redirect developmental pathways, and increase susceptibility to CKD later in life [76]. Key mechanisms implicated in kidney programming include oxidative stress, epigenetic modifications, impaired NO signaling, dysregulation of the renin–angiotensin system (RAS), disturbances in nutrient-sensing pathways, gut microbiota dysbiosis, and inflammation [2,3,34,35].

In humans, full-term neonates typically possess approximately one million nephrons per kidney, although this number can vary more than tenfold [6,77]. Nephrons are generated primarily through branching morphogenesis of the ureteric bud during fetal development. Adverse prenatal or perinatal conditions—such as intrauterine growth restriction (IUGR), preterm birth, maternal or neonatal malnutrition, or exposure to nephrotoxic medications like gentamicin—may disrupt this process and reduce nephron endowment, potentially leading to congenital anomalies of the kidney and urinary tract (CAKUT) [78,79].

Reduced nephron number represents a fundamental “first hit” in kidney programming, limiting renal reserve and rendering remaining nephrons more susceptible to secondary insults throughout life [5,6]. Because direct mechanistic studies in human neonates are constrained by ethical and technical limitations, much of the mechanistic understanding of kidney programming derives from experimental animal models, although a key translational limitation is that rodent nephrogenesis extends into the early postnatal period, whereas in humans it is largely completed in utero, leading to differences in developmental timing and exposure windows.

### 5.2. Kidney Programming: The Interplay Between Maternal Gut Dysbiosis and Oxidative Stress

#### 5.2.1. Maternal Insults as Triggers of Oxidative Stress-Mediated Kidney Programming

Evidence from experimental models supports oxidative stress as a central mechanism in kidney programming. A range of maternal insults—including nutritional imbalance [80,81], maternal disease [82,83], environmental toxicants [84,85], and medication exposure [86]—can disrupt fetal redox homeostasis during critical windows of nephrogenesis, leading to persistent structural and functional renal alterations and increased lifetime risk of CKD and hypertension.

Among these, maternal nutritional perturbations are the most extensively studied, with both deficiency and excess (e.g., protein restriction, high-fat or high-fructose diets, and micronutrient imbalance) inducing oxidative stress-mediated renal programming in offspring [87]. These conditions disrupt redox balance (ROS/RNS), impair nephron maturation, and permanently alter renal structure and function.

Maternal diseases such as diabetes, hypertension, CKD, and inflammation also promote oxidative stress-mediated programming through systemic endothelial dysfunction and placental impairment [87]. Environmental exposures (e.g., pollutants, heavy metals, endocrine disruptors, and smoking) further exacerbate fetal oxidative stress, contributing to reduced nephron endowment and long-term renal vulnerability [85].

#### 5.2.2. Molecular Mechanisms in Oxidative Stress-Induced Kidney Programming

The developing fetus is highly susceptible to oxidative injury due to immature antioxidant defense systems [44], limiting the capacity to neutralize excess ROS and RNS. Under adverse intrauterine conditions, excessive ROS/RNS can overwhelm redox buffering capacity, leading to damage to proteins, lipids, and DNA.

These mechanisms include upstream processes that increase ROS generation—such as upregulated expression of ROS-producing enzymes and enhanced ROS synthesis—followed by secondary downstream effects, including increased peroxynitrite formation, depletion of antioxidant capacity, elevated ADMA, reduced NO bioavailability, and accumulation of oxidative damage [87]. It also induces epigenetic modifications, including altered DNA methylation, histone modifications, and microRNA regulation [88], which may lead to persistent changes in gene expression affecting RAS signaling, nutrient sensing, mitochondrial function, and inflammation. These mechanisms contribute to long-term structural and functional renal alterations, including reduced nephron endowment, glomerulosclerosis, tubular injury, and albuminuria, increasing susceptibility to hypertension and CKD in adulthood [89].

In rodents, where nephrogenesis extends into the postnatal period, both prenatal and early postnatal exposures influence renal development, providing a broader window to study kidney programming and potential preventive interventions [90].

#### 5.2.3. Maternal Gut Microbiota in the Developmental Origins of Kidney Disease

In parallel with oxidative stress, accumulating evidence indicates that maternal gut microbiota plays an important role in shaping the intrauterine and early-life environments that influence kidney programming [19,20]. The maternal gut microbiome functions as a metabolically active ecosystem that interacts with host physiology through the production of microbial metabolites, modulation of immune responses, and regulation of systemic metabolic pathways. Disruptions in this microbial community—commonly referred to as gut dysbiosis—can therefore have systemic consequences that extend beyond the gastrointestinal tract. During pregnancy, physiological changes in maternal metabolism and immunity are accompanied by shifts in gut microbial composition, and disturbances in this process may influence maternal–fetal interactions.

Maternal gut dysbiosis can affect host metabolism, immune responses, and redox balance through the production of microbial metabolites and inflammatory mediators. Changes in microbial composition can alter the generation of bioactive compounds such as SCFAs, TMAO, bile acid derivatives, and other microbial metabolites that influence host signaling pathways. These metabolites can enter the maternal circulation and potentially affect placental function and fetal development. SCFAs, for instance, are known to regulate energy metabolism, immune responses, and oxidative stress pathways, whereas excessive levels of certain microbial metabolites may contribute to metabolic disturbances and inflammatory activation. Through these mechanisms, maternal gut dysbiosis may amplify oxidative stress and inflammatory signaling within the maternal–placental–fetal axis.

#### 5.2.4. Experimental Evidence Linking Gut Dysbiosis to Kidney Programming

Consistent with this concept, several experimental models of developmental renal disease demonstrate concurrent alterations in maternal or offspring gut microbiota alongside oxidative stress and kidney dysfunction. Models involving maternal high-fructose diets [91], high-fat diets [92], toxin exposure [93], and maternal CKD [94] have all been associated with shifts in gut microbial composition and metabolic profiles. In many of these models, microbial dysbiosis coexists with increased oxidative stress, altered metabolite production, and impaired renal development. These findings suggest that the gut microbiota may function as an intermediary linking environmental exposures to redox imbalance and kidney developmental abnormalities.

The interaction between microbial metabolism and host oxidative pathways therefore represents a critical component of the gut–kidney axis during early life. Microbiota-derived metabolites can influence oxidative signaling pathways, while oxidative stress itself may further alter microbial community composition, creating a bidirectional relationship between host physiology and the gut microbiome. Disruption of this delicate balance during critical developmental periods may have lasting effects on renal structure and function. A deeper understanding of how microbial metabolism interacts with host redox signaling during sensitive developmental windows may provide valuable insights into the developmental origins of kidney disease. Such knowledge may also open new opportunities for early-life interventions—including nutritional modulation, microbiota-targeted therapies, and antioxidant strategies—aimed at preventing CKD and its associated cardiometabolic complications across the life course.

### 5.3. Childhood: The Foundation of the Gut–Redox Axis

During early childhood, the gut microbiota functions as a critical microbial organ that supports immune maturation and metabolic homeostasis [95]. Initial colonization—primarily through vertical transmission from the mother—establishes pioneer microbial communities essential for long-term health [17]. Beneficial taxa such as *Bifidobacterium* and *Lactobacillus* contribute to immune balance and antioxidant defenses. However, disruptions during this plastic period—such as cesarean delivery, early antibiotic exposure, or maternal environmental enteric dysfunction—may lead to persistent dysbiosis.

Such early microbial disturbances may influence epigenetic regulation and immune development, potentially lowering the threshold for systemic inflammation. In childhood, these changes may manifest as subtle alterations in intestinal permeability and reduced production of SCFAs, metabolites that help maintain mitochondrial integrity and suppress renal inflammation [58].

Clinical studies increasingly suggest that disturbances in the gut–redox axis also occur in pediatric CKD [96,97]. Children with early-stage CKD often show reduced microbial diversity and shifts toward dysbiosis, including decreased abundance of SCFA-producing taxa such as *Prevotella* and increased representation of potentially pro-inflammatory bacteria including members of the *Enterobacteriaceae* and *Enterococcaceae* families [96,97,98]. Dysregulated microbial metabolism may alter production of metabolites such as TMAO and protein-bound uremic toxins. In pediatric CKD cohorts, microbial alterations have been associated with reduced urinary TMAO levels but potentially increased circulating concentrations as renal clearance declines [98].

Consistent with these microbial and metabolic disturbances, children with CKD often exhibit elevated oxidative stress biomarkers even at early disease stages [99]. Increased lipid peroxidation markers such as malondialdehyde correlate with disease severity and cardiovascular risk indicators including left ventricular hypertrophy and arterial stiffness [100]. These findings suggest that disruption of the gut–redox balance may contribute to both renal injury and early cardiovascular complications in pediatric CKD.

### 5.4. Adulthood: Cumulative Stressors and the Gut–Redox Feedback Loop

During adulthood, cumulative environmental, metabolic, and lifestyle exposures continue to shape the gut–kidney axis. Cardiovascular–kidney–metabolic (CKM) syndrome, a systemic clinical condition characterized by intertwined pathological and physiological interactions among metabolic abnormalities, CKD, and CVD, further exemplifies the integrated nature of cardiometabolic and kidney dysfunction leading to multi-organ impairment and increased cardiovascular risk [101]. Within this context, metabolic disorders—including obesity, diabetes, and hypertension—along with dietary habits and environmental toxicants, can disrupt gut microbial composition and sustain oxidative stress [102,103]. As in children, growing evidence shows that adults with CKD exhibit reduced microbial diversity and depletion of beneficial commensal bacteria such as *Bifidobacterium*, *Lactobacillus*, and *Prevotella*, accompanied by expansion of opportunistic taxa including members of the *Enterobacteriaceae* family [104,105]. This dysbiotic shift favors proteolytic fermentation and increased production of microbial metabolites rather than beneficial SCFAs [16].

Among these metabolites, protein-bound uremic toxins—including IS and pCS—as well as TMAO play key roles in mediating oxidative stress and inflammation [106,107]. These compounds exert distinct but complementary pathogenic effects: IS primarily enters renal tubular cells via OTAs, where it activates NF-κB signaling and promotes oxidative stress and mitochondrial dysfunction, whereas TMAO predominantly targets endothelial cells, contributing to endothelial dysfunction, vascular inflammation, and cardiovascular risk; pCS similarly induces oxidative stress and inflammatory signaling in renal and vascular tissues. Damage to intestinal epithelial tight junctions by oxidative stress increases intestinal permeability, allowing endotoxins and microbial metabolites to enter the systemic circulation. As kidney function declines, elevated urea levels in the intestinal lumen promote the growth of urease-producing bacteria, further aggravating dysbiosis. Through these mechanisms, renal dysfunction and microbial imbalance reinforce each other, forming a bidirectional gut–kidney feedback loop that accelerates CKD progression [108,109].

Oxidative stress and inflammation also damage intestinal tight junction proteins, increasing intestinal permeability and allowing microbial products and endotoxins to enter the circulation. As renal function declines, elevated urea levels in the intestinal lumen further alter the microbial environment by promoting the growth of urease-producing bacteria. Through these interconnected processes, kidney dysfunction and gut dysbiosis reinforce one another, establishing a bidirectional gut–kidney feedback loop that contributes to progressive renal injury and cardiovascular complications during adulthood.

### 5.5. Aging: Loss of Redox Resilience and Advanced Kidney Vulnerability

Aging is characterized by a progressive decline in the kidney’s capacity to maintain redox and metabolic homeostasis, resulting in increased vulnerability to injury [110,111]. Structural and functional changes accumulate over time, including nephron loss, glomerulosclerosis, tubular atrophy, and interstitial fibrosis, collectively reducing renal reserve. These processes are accompanied by mitochondrial dysfunction and increased ROS production, while age-related impairment of antioxidant defenses further limits the capacity to counteract oxidative damage. Reduced activity of protective pathways, including Klotho signaling and Sirtuin-mediated stress responses, also contributes to diminished cellular resilience and impaired repair [112,113].

Concurrently, aging is associated with altered gut microbial composition, including reduced diversity and depletion of SCFA-producing bacteria [114]. Decreased SCFA availability may impair intestinal barrier integrity, mitochondrial function, and systemic inflammatory control, thereby amplifying oxidative stress. In CKD, these age-related alterations are further exacerbated, with accumulation of gut-derived uremic toxins and persistent inflammation promoting endothelial dysfunction, vascular calcification, and cardiac remodeling.

Overall, aging represents a convergence of lifelong environmental exposures, cumulative oxidative injury, and progressive alterations in renal structure and gut microbiota, increasing susceptibility to kidney failure and cardiovascular complications [115]. Understanding the evolution of the gut–redox axis across the life course may provide a framework for strategies aimed at preserving renal resilience and reducing CKD burden.

## 6. Early Prevention via Redox–Gut Axis Reprogramming

Antioxidant therapies and gut microbiota-targeted interventions have demonstrated clear benefits in established CKD, as extensively reviewed elsewhere [116,117,118,119]. However, human evidence for their potential to prevent kidney disease by modulating the redox–gut axis during early life remains limited. Importantly, the translational therapeutic window must be interpreted in a species-specific manner: whereas nephrogenesis extends into the postnatal period in rodents, it is largely completed before birth in humans. Therefore, interventions effective in postnatal rodent models likely correspond to prenatal—particularly early to mid-gestational—windows in human development, when nephron induction, branching morphogenesis, and microvascular maturation are most active. Emerging experimental studies indicate that interventions targeting oxidative stress and gut microbial composition during critical developmental windows may offer a promising preventive strategy. In animal models, approaches such as antioxidant supplementation, probiotics, prebiotics, or dietary modifications have been shown to attenuate adverse renal outcomes induced by early-life insults, as illustrated by representative examples in Table 1 [80,85,91,92,94,120,121,122,123,124,125,126,127,128,129,130,131,132,133]. These results support the concept that early-life modulation of the redox–gut axis can reprogram renal development, enhance kidney resilience, and reduce susceptibility to CKD in later life.

### 6.1. Natural Antioxidants

Several natural antioxidants, including vitamins, amino acids, melatonin, and polyphenol, during pregnancy and lactation have shown benefits to kidney health and prevent kidney programming.

Vitamins C and E are the most commonly used antioxidants: vitamin C, a water-soluble free radical scavenger, and vitamin E, a lipid-soluble inhibitor of oxidative enzymes [134,135]. Vitamins C and E, among others, support kidney health [136], and maternal vitamin C supplementation protects offspring from LPS-induced hypertension by reducing intrarenal oxidative stress and modulating the RAS [120]. However, high doses of vitamins A, β-carotene, and E have been linked to increased mortality [137]; therefore, perinatal vitamin supplementation should be used cautiously, only in cases of deficiency, with attention to contamination risks for the developing fetus [138].

Certain dietary amino acids with antioxidant properties exert therapeutic and protective effects in kidney diseases [139,140]. In light of evidence that NO deficiency contributes to the developmental origins of kidney disease, perinatal supplementation with the NO-related amino acids arginine and citrulline has been investigated as a potential intervention to prevent adult-onset renal pathology in the offspring [80,121,122]. Arginine serves as the substrate for NOS-mediated NO production, whereas citrulline is a precursor of arginine [141,142]. The human kidney can convert citrulline to arginine [143]. Moreover, oral citrulline supplementation bypasses hepatic metabolism, thereby enhancing arginine production and increasing NO bioavailability [143]. Accordingly, citrulline has been more commonly used as a supplement than arginine to enhance NO bioavailability and protect adult rat offspring from kidney programming in oxidative stress-related models, as reviewed elsewhere [87].

In addition, tryptophan, taurine, and cysteine have also been evaluated as reprogramming interventions targeting oxidative stress in maternal CKD-induced kidney programming models [123,124,125]. Despite evidence that glycine and branched-chain amino acids can improve kidney outcomes [144], the role of oxidative stress reduction in mediating these protective effects has not been fully elucidated.

Another natural antioxidant is melatonin [145]. Melatonin, an endogenous tryptophan-derived indolamine, exerts multiple biological functions [146]. It has been clinically applied as an antioxidant therapy in pregnant women, neonates, and children [147,148,149]. Melatonin also plays an important role in pregnancy and fetal development [150]. Melatonin, along with its breakdown products, counteracts ROS and RNS, upregulates antioxidant systems, and enhances nitric oxide availability [151]. Experimental data demonstrate that perinatal melatonin administration may prevent adult-onset conditions, including kidney disorders [152]. As a potent antioxidant, melatonin administration during gestation and lactation has been shown to protect adult rat offspring against maternal caloric restriction-induced kidney programming and hypertension [126]. Its reprogramming effects include reduced renal 8-OHdG expression, restored NO bioavailability, and modulation of the RAS, another important source of oxidative stress in kidney disease. Additionally, maternal melatonin treatment has demonstrated renoprotective effects in several oxidative stress-related kidney programming models, including maternal methyl donor diet, L-NAME exposure, and high fructose intake, as reviewed elsewhere [87]. Although melatonin has a favorable safety profile [153,154], its routine use during pregnancy is not currently recommended [155]. Therefore, the perinatal use of melatonin as a preventive strategy for kidney health, particularly in fetuses and neonates, awaits further clinical translation.

Resveratrol is another natural antioxidant with renoprotective properties [156,157]. It is a naturally occurring nonflavonoid polyphenol belonging to the stilbene family [158]. Resveratrol exerts antioxidant effects by scavenging ROS/RNS, enhancing antioxidant enzyme activity, increasing glutathione levels, and upregulating NOS expression [159]. Perinatal administration of resveratrol has been shown to protect adult rat offspring from maternal CKD-induced kidney programming and hypertension [94]. Beyond its antioxidant activity, resveratrol may also act as a prebiotic by restoring microbial richness, increasing beneficial genera such as *Lactobacillus* and *Bifidobacterium*, and modulating microbial metabolite pathways [94]. As reviewed elsewhere [87], beneficial effects of resveratrol on renal outcomes in adult offspring have been demonstrated in several rat models of kidney programming, including maternal high-fructose diet, maternal NO deficiency, bisphenol A exposure, and TCDD exposure. However, a major limitation of the clinical utility of polyphenols is their low bioavailability [160]. Considering the substantial interindividual variability and complexity of resveratrol pharmacokinetics, future studies are needed to improve its therapeutic efficacy. Advanced delivery systems and artificial intelligence–assisted optimization strategies may provide promising approaches to enhance therapeutic precision and scalability [161].

### 6.2. Synthetic Antioxidants

Along with natural antioxidants, several synthetic antioxidants have also been investigated for their therapeutic potential in kidney diseases [24]. MitoQ, a mitochondria-targeted analogue of coenzyme Q10, reduces oxidative stress by suppressing superoxide production and lipid peroxidation [162]. A previous study demonstrated that perinatal MitoQ treatment prevented hypertension in adult mouse offspring, reduced mitochondrial oxidative stress, and attenuated structural and functional kidney abnormalities in a maternal smoking model [85].

N-acetylcysteine (NAC) is a well-known synthetic antioxidant, although it is derived from the natural amino acid cysteine [163]. NAC serves as a precursor of glutathione and a cysteine analogue that can be utilized for hydrogen sulfide (H_2_S) synthesis [164]. Perinatal NAC therapy prevented hypertension and oxidative stress in adult rat offspring exposed to maternal suramin administration, which was associated with increased glutathione production, restoration of NO bioavailability, and augmentation of the H_2_S signaling pathway [127].

Dimethyl fumarate (DMF), a well-established Nrf2 activator [165], provides another example of redox-targeted intervention. In a model combining prenatal dexamethasone exposure with a postnatal high-fat diet, DMF conferred protection by lowering oxidative stress markers—including ADMA and 8-OHdG—while simultaneously increasing NO levels [128].

Synthetic antioxidants, such as SOD mimetics, have also demonstrated potential in managing oxidative stress-related disorders [166]. For instance, gestational administration of the SOD mimetic tempol reduced proteinuria and BP in adult offspring of spontaneously hypertensive rats [167]. Despite these encouraging preclinical results, none of these agents have yet been applied in clinical maternal–fetal or neonatal care.

### 6.3. Gut Microbiota-Targeted Interventions

Early-life gut microbiota-targeted interventions—including probiotics, prebiotics, postbiotics, and modulation of microbial metabolites—have been investigated in various animal models of kidney programming. In clinical practice, the most commonly used microbiome-modulating approaches are probiotics and prebiotics. Probiotics are defined as live microorganisms that provide health benefits when consumed in sufficient amounts [168], while prebiotics are substrates that selectively stimulate the growth and activity of beneficial gut bacteria [168]. Synbiotics combine both strategies. Although these interventions may reduce uremic toxin production, robust clinical evidence for CKD prevention or treatment in humans remains limited [23,109,169,170].

As summarized in Table 1, preclinical studies consistently demonstrate protective effects of microbiota modulation on BP and renal outcomes in developmental programming models. For example, *Lactobacillus casei* protects against hypertension induced by maternal high-fructose or high-fat diets, likely via modulation of gut microbiota composition, restoration of SCFA signaling, and regulation of nutrient-sensing pathways [91,92]. Similarly, prebiotic interventions such as inulin reshape microbial composition, increase SCFA production, and are associated with improved BP control and renal protection in experimental models [91,92].

Beyond classical probiotics and prebiotics, dietary and phytochemical interventions (e.g., resveratrol and garlic oil) also exert microbiome-mediated protective effects [171]. Resveratrol restores microbial diversity and suppresses inflammatory signaling (including AhR- and Th17-related pathways) [94], while garlic oil increases SCFA-producing bacteria and enhances hydrogen sulfide (H_2_S) signaling via upregulation of H_2_S-generating enzymes [129]. Collectively, these findings highlight H_2_S as a key mechanistic link between gut microbiota and oxidative stress in kidney programming, acting through antioxidant and Nrf2-related pathways [172,173,174,175]. These interventions prevent hypertension and kidney injury by restoring H_2_S signaling and reshaping the gut microbiome. For example, maternal NAC increases fecal thiosulfate and *Bifidobacterium* abundance, mitigating oxidative damage induced by gut dysbiosis during critical developmental windows in spontaneously hypertensive rats [176].

Postbiotics, defined as microbial metabolites or components with biological activity, represent an emerging alternative to live microbial therapy, particularly in vulnerable populations where probiotic safety may be a concern [177,178,179]. However, very limited information is available regarding the use of postbiotics in human CKD [180]. Emerging evidence supports the potential of SCFAs as postbiotic agents for CVD prevention [181]. Among these, short-chain fatty acids (SCFAs; acetate, propionate, and butyrate) have been most extensively studied. In these models, SCFAs exert protective effects primarily through activation of SCFA-mediated signaling pathways, including increased renal expression of SCFA receptors, modulation of gut microbiota–derived metabolites, and receptor-dependent regulation of vascular tone and the RAS [130,131,182]. In particular, butyrate may also enhance nitric oxide bioavailability, supporting its potential antioxidant and vasoprotective effects [183].

Another microbiota-targeted therapy is fecal microbiota transplantation (FMT). Although FMT has been extensively studied in microbiome-related diseases [184,185], its application in human CKD remains limited to preclinical and preliminary clinical studies [186,187]. Only one study has evaluated FMT in a kidney programming model, where maternal FMT from healthy donors attenuated high-fructose-induced programmed hypertension in offspring, accompanied by increased plasma butyrate, upregulation of SCFA receptors (e.g., GPR41, GPR43), reduced oxidative stress, and restoration of gut microbiota–metabolite homeostasis [132].

Other microbiome-modulating approaches include the oral intestinal sorbent AST-120, which reduces microbiota-derived uremic toxins [188] and has shown cardiovascular benefits in adult CKD patients [189], although its effects on gut microbiota composition and kidney programming remain unclear.

In addition, microbial TMAO production can be inhibited by targeting gut microbial choline TMA lyase using 3,3-dimethyl-1-butanol (DMB) or iodomethylcholine (IMC) [190,191]. Maternal inhibition of TMAO formation with DMB or IMC prevents offspring hypertension in maternal CKD and high-fructose diet models, respectively [130,133], through suppression of the TMA–TMAO pathway and remodeling of the gut microbiota.

Collectively, these findings support the concept of developmental reprogramming of the gut–redox axis, whereby early microbiota-targeted interventions may redirect long-term renal and cardiovascular trajectories. However, translation to human CKD prevention remains an important unmet challenge requiring further clinical validation.

## 7. Conclusions and Future Perspective

The DOHaD framework reframes CKD as a consequence of developmental programming rather than solely the cumulative result of renal insults acquired during adulthood. Although this paradigm has substantially advanced our understanding of early-life determinants of renal vulnerability, key conceptual and methodological challenges remain, particularly regarding the gut–redox axis.

A central unresolved issue is the bidirectional relationship between gut dysbiosis and oxidative stress in CKD, which may function as both cause and consequence of uremia-associated barrier dysfunction, systemic inflammation, and microbiota-derived toxin accumulation [18,97,192]. This ambiguity is compounded by limitations in current microbiome research and model systems, where substantial variability in experimental and clinical conditions hampers causal inference and translational robustness [193].

Future progress will require more physiologically faithful platforms, such as multi-organ microphysiological systems, to better reproduce gut–kidney–immune interactions under controlled conditions [194,195]. In parallel, priority should be given to identifying microbiota-derived metabolites that regulate host redox signaling pathways (e.g., Nrf2, NF-κB, mitochondrial ROS, and inflammasome activation) as potential mechanistic links and therapeutic targets [196].

Another critical gap involves understanding how oxidative stress disrupts intestinal barrier integrity and promotes the “leaky gut–renal inflammation” loop through endotoxin translocation and systemic immune activation. This complexity is further underscored by the “antioxidant paradox,” whereby certain antioxidants may exhibit prooxidant activity depending on cellular redox conditions, acting as modulators rather than uniformly protective agents [197]. Within the DOHaD paradigm, it is also unclear whether early-life perturbations of gut–redox balance can impair nephron development and thereby predispose individuals to CKD later in life. In addition, host genetic variability in antioxidant defense systems and immune signaling pathways may modify susceptibility to microbiota-driven oxidative injury, highlighting the importance of host–microbiome interactions and suggesting that antioxidant effects may be context-dependent across developmental stages.

From a translational perspective, determining whether modulation of the gut–redox axis through microbiota-targeted therapies, dietary interventions, or reduction in intestinal toxin generation can alter CKD trajectory remains an important unresolved question. The identification of reliable gut-derived metabolites and oxidative stress-related markers as predictive biomarkers may further facilitate precision nephrology approaches.

Collectively, future studies must address a central question: how the gut microbiota regulates systemic redox homeostasis and thereby influences the initiation, progression, and potential prevention of CKD across the life course.

## Figures and Tables

**Table 1:** Selected examples of early-life redox–gut axis reprogramming therapies for kidney programming-related offspring comorbidities.

Intervention	Dose/Period	Animal Model	Effect	Ref.
Natural Antioxidants				
Vitamin C	350 mg/kg/day i.p. daily /GD 8 to 14	Prenatal LPS Exposure, SD rat/M	Prevented hypertension, mitigated intrarenal oxidative stress, reversed epigenetic alterations, specifically histone H3 acetylation, on the ACE1 promoter to normalize ACE1 expression	[120]
Arginine, taurine, Vitamins C and E	2 weeks before until 8 weeks after birth	Genetic hypertension, SHR/M and F	Prevented hypertension, enhanced NO bioavailability, and decreased renal oxidative stress	[121]
Arginine, taurine, Vitamins C and E	2 weeks before until 4 weeks after birth	Genetic hypertension, FHH rat/M and F	Reduced blood pressure, proteinuria, glomerulosclerosis, and enhanced NO bioavailability	[122]
Citrulline	0.25% in drinking water during/G+L	Maternal caloric restriction diet (50%), SD rat/M	Prevented reduction in nephron numbers, glomerular hypertrophy, the rise in plasma creatinine, and restored NO bioavailability	[80]
Tryptophan	200 mg/kg body weight /day via oral gavage/G+L	Maternal adenine-induced CKD, SD rat/M	Prevented hypertension, restored NO bioavailability, shaped gut microbiota profile, modulated AhR signaling, and rebalanced the RAS	[123]
Taurine	3% in drinking water/G+L	Maternal adenine-induced CKD, SD rat/M	Prevented hypertension, augmented the hydrogen sulfide pathway, rebalanced the RAS, and modulated the gut microbiota	[124]
Cysteine	8 mmol/kg body weight/day/G	Maternal adenine-induced CKD, SD rat/M	Prevented hypertension, enhanced hydrogen sulfide production, restored NO bioavailability, rebalanced the RAS, and reshaped the gut microbiota	[125]
Melatonin	0.01% in drinking water/G+L	Maternal caloric restriction, SD rat/M	Prevented hypertension, reduced renal 8-OHdG expression, restored NO bioavailability, modulated the RAS, and induced long-term epigenetic changes in renal gene expression.	[126]
Resveratrol	50 mg/L in drinking water/G+L	Maternal adenine-induced CKD, SD rat/M	Prevented hypertension, improved NO bioavailability, reducing renal oxidative stress, restored microbial richness, increased *Lactobacillus* and *Bifidobacterium*, and modulated microbial metabolite pathways (TMAO and SCFA signaling).	[94]
Synthetic Antioxidants				
MitoQ	500 μM in drinking water/G+L	Maternal smoking exposure, Balb/c mice/M	Prevented hypertension, reduced mitochondrial oxidative stress, and prevented structural and functional kidney abnormalities.	[85]
N-acetylcysteine (NAC)	1% in drinking water/G+L	Maternal suramin-induced preeclampsia, SD rat/M	Prevented, hypertension, restored NO and H_2_S signaling, increased antioxidant glutathione levels, and normalized renal H_2_S-synthesizing enzyme activity.	[127]
Dimethyl fumarate	50 mg/kg body weight/day via gastric gavage/G	Prenatal dexamethasone and postnatal high-fat diet	Prevented hypertension, activated Nrf2 signaling, reduced renal oxidative stress, rebalanced the RAS, and promoted renal autophagy	[128]
Gut Microbiota-Targeted Interventions				
*Lactobacillus casei*	2 × 108 CFU/day via gavage /G+L	Maternal high-fructose diet, SD rat/M	Prevented hypertension, modulated gut microbiota, regulated SCFA signaling, and restored nutrient-sensing signaling pathway.	[91]
Inulin	5% w/w/G+L	Perinatal high-fat diet, SD rat/M	Prevented hypertension, reshaped gut microbiota, modulated microbial metabolites (SCFAs and TMAO), and regulated the RAS	[92]
Garlic oil	100 mg/kg/day via gavage/G+L	Perinatal high-fat diet, SD rat/M	Prevented hypertension, increased alpha-diversity and beneficial bacteria (*Lactobacillus*, *Bifidobacterium*), enhanced renal H_2_S-generating enzyme activity, and increasing NO bioavailability	[129]
Acetate	200 mmol/L in drinking water/G+L	Maternal high-fructose diet, SD rat/M	Prevented hypertension, and modulated microbial metabolites (SCFAs and TMAO)	[130]
Butyrate	400 mg/kg body weight/day in drinking water/G+L	Maternal high-fructose diet, SD rat/M	Prevented hypertension, improved TMAO metabolism, and enhanced NO bioavailability	[131]
Propionate	200 mmol/L in drinking water/G+L	Maternal high-fructose diet, SD rat/M	Prevented hypertension and modulated gut microbiota composition	[131]
Fecal microbiota transplantation (FMT)	Received FMT from ND donors on GD 1, 7, and 14	Maternal high-fructose diet, SD rat/M	Prevented hypertension, restored gut microbiota composition, increased plasma SCFAs and SCFA receptor expression, and reduced renal oxidative stress	[132]
Iodomethylcholine	0.06% w/w in chow /G+L	Maternal adenine-induced CKD, SD rat/M	Prevented hypertension, reduction in TMAO levels, attenuation of oxidative stress, and remodeling of gut microbiota composition.	[133]
3,3-dimethyl-1-butanol	1% in drinking water/G+L	Maternal high-fructose diet, SD rat/M	Prevented hypertension, and modulated microbial metabolites (SCFAs and TMAO)	[130]

G: gestation; L: lactation; NO: nitric oxide; RAS: renin-angiotensin system; SD: Sprague–Dawley; SHR: spontaneously hypertensive rat; FHH: Fawn-hooded hypertensive rat; CKD: chronic kidney disease; LPS: lipopolysaccharide; SCFA: short-chain fatty acid; TMAO: trimethylamine-N-oxide; M: male; F: female.

## Data Availability

No new data were created or analyzed in this study.
